# Tofacitinib as a Rescue Therapy for Ulcerative Colitis in Pregnancy

**DOI:** 10.1093/ibd/izae076

**Published:** 2024-04-09

**Authors:** Catherine Rowan, Fiona Yeaman, Kenneth Ernest-Suarez, Marie-Louise Martin, Remo Panaccione, Tushar Shukla, Matt Grossi, Meghan Vlasschaert, Kerri Novak, Cynthia Seow

**Affiliations:** Inflammatory Bowel Disease Unit, Division of Gastroenterology and Hepatology, University of Calgary, Calgary, Canada; Inflammatory Bowel Disease Unit, Division of Gastroenterology and Hepatology, University of Calgary, Calgary, Canada; Inflammatory Bowel Disease Unit, Division of Gastroenterology and Hepatology, University of Calgary, Calgary, Canada; Inflammatory Bowel Disease Unit, Division of Gastroenterology and Hepatology, University of Calgary, Calgary, Canada; Inflammatory Bowel Disease Unit, Division of Gastroenterology and Hepatology, University of Calgary, Calgary, Canada; Inflammatory Bowel Disease Unit, Division of Gastroenterology and Hepatology, University of Calgary, Calgary, Canada; Obstetrics and Gynecology, Rockyview General Hospital, University of Calgary, Calgary, Canada; General Internal Medicine and Obstetric Medicine, Educational Director, OBIM, University of Calgary, Calgary, Canada; Inflammatory Bowel Disease Unit, Division of Gastroenterology and Hepatology, University of Calgary, Calgary, Canada; Inflammatory Bowel Disease Unit, Division of Gastroenterology and Hepatology, University of Calgary, Calgary, Canada

**Keywords:** ulcerative colitis, pregnancy, tofacitinib

## Case Report

A 28-year-old G1P0 female with pancolitis (5 years duration) maintained on 90 mg of ustekinumab q4 weekly with prior secondary loss of response to infliximab was referred with a disease flare. Despite endoscopic remission 3 months prior to conception, by 8 weeks gestation, she was symptomatic, fecal calprotectin was >10 000 µg/g, and intestinal sonography demonstrated increased left colonic wall thickness and hyperemia. Oral corticosteroids and 5-ASA suppositories were commenced. Inability to wean prednisone <20 mg daily necessitated ustekinumab reinduction. Despite this, sigmoidoscopy at 15 weeks’ gestation demonstrated a Mayo 2 subscore, hence 10 mg of tofacitinib twice daily was commenced. Prophylactic measures including the nonlive Shingrix vaccination, 81 mg of aspirin for pre-eclampsia prophylaxis, and tinzaparin to address the combined thrombotic risk factors of active IBD, pregnancy, and tofacitinib were provided as per gastroenterology, materno-fetal medicine, and internal medicine input.^1,2^ Corticosteroid-free clinical and sonographic remission (Table 1) was achieved at 34 weeks’ gestation with ongoing 10 mg of tofacitinib twice daily, ustekinumab q4 weekly, and 5-ASA suppositories.

**Table 1.** Sequential intestinal ultrasounds performed during pregnancy showing interval improvement and normalisation of parameters through pregnancy.

Gestation	IUS Parameters	Rectum	Sigmoid Colon	Descending Colon	Transverse Colon	Ascending Colon	Terminal Ileum
14 weeks	BWT (mm)	No data	4.8	4.3	2.2	2.1	1.9
	LS		2	2	0	0	0
28 weeks	BWT (mm)	No data	2.7	5.0	3.0	<3	<3
	LS		0	1	0	0	0
35 weeks	BWT (mm)	3.7	2.2	2.7	1.2	2.0	No data
	LS	0	0	0	0	0	

Abbreviations: IUS, intestinal ultrasound; BWT, bowel wall thickness (normal ≤ 3 mm); LS, Limberg score measure of hyperemia (≥2 abnormal).

Serial obstetric ultrasounds demonstrated appropriate fetal biometry. Caesarean section at 39 weeks’ gestation ensued due to development of variable complex decelerations on tocography. Birthweight was 2920 grams. There were no congenital anomalies. Neonatal ICU admission was not required.

Tofacitinib at 10 mg twice daily was discontinued at delivery due to anticipated breastfeeding. Postpartum course was complicated by maternal COVID-19 infection and mastitis but in the presence of ongoing remission (calprotectin 145 µg/g) with ustekinumab and 5-ASA suppositories. The infant received all 2-month scheduled vaccinations without complications.

## Discussion

Tofacitinib with its quick onset and rapid clearance^3^ was used in the second and third trimesters of pregnancy, as rescue therapy in the setting of active steroid-dependent UC despite optimized biologic and rectal 5-ASA therapy. Caution exists using tofacitinib during the critical first trimester of fetal organogenesis and while breastfeeding. Animal studies demonstrated feticidal and teratogenic effects with doses exceeding the maximum recommended human dose.^4^ However, limited human clinical trial^5,6^ and postmarketing data demonstrated spontaneous abortion and congenital malformation rates similar to the general population.^7^ Julsgaard reported detectable tofacitinib milk concentrations supporting transmammary transfer.^8^

Tofacitinib led to avoidance of surgery, facilitated corticosteroid-free remission, and helped with term delivery of a healthy infant. Potential risks of herpes zoster and VTE were minimized with Shingrix vaccination and anticoagulation.^2,3^ Sequential noninvasive monitoring, thoughtful collaboration with the patient, IBD team, maternofetal medicine, and internal medicine were instrumental in navigating this difficult but ultimately successful clinical scenario.^9,10^
